# The attitudes toward hookah smoking in the MENA region scale (ATHS-MENA): factor analysis and validation

**DOI:** 10.3389/fpsyg.2026.1747480

**Published:** 2026-09-17

**Authors:** Gabriel Andrade, Abderrahim Benlahcene, Enas Mohamed, Dalia Bedewy

**Affiliations:** 1 Department of Basic Sciences, College of Medicine, Ajman University, Ajman, United Arab Emirates; 2 Department of Psychology, College of Humanities and Sciences, Ajman University, Ajman, United Arab Emirates; 3 Department of Educational Psychology, Faculty of Education, Mansoura University, Mansoura, Egypt; 4 Department of Educational Psychology, Faculty of Education, Tanta University, Tanta, Egypt

**Keywords:** hookah smoking, MENA region, reliability, smoking behavior, validity

## Abstract

**Background:**

Hookah smoking poses a global health challenge, especially in the MENA region where it holds deep cultural significance. Existing smoking scales often lack the cultural nuances needed for accurate assessment. This study aimed to develop and validate a culturally relevant instrument, the Attitudes Toward Hookah Smoking Scale in the MENA region (ATHS-MENA).

**Methods:**

The ATHS-MENA was developed through a systematic process including item generation, expert review, cognitive interviewing, and pilot testing. The scale was administered to participants in the UAE. Exploratory factor analysis (EFA) identified the factor structure, followed by confirmatory factor analysis (CFA) to assess model fit. Convergent validity was tested against an existing smoking attitude scale. Known-groups validity was examined based on gender, nationality, Emirati status, and smoking behavior.

**Results:**

EFA revealed four factors: Hookah Engagement/Dependency, Social/Cultural Acceptance, Avoidance/Health Awareness, and Risk Minimization. The scale demonstrated high internal reliability (α = 0.94). Inter-factor correlations ranged from −0.41 to 0.57, with the four-factor solution explaining 68.4% of total variance. CFA indicated acceptable model fit following *post-hoc* modifications (RMSEA = 0.079, CFI = 0.912, TLI = 0.901). Convergent validity was established with the Smoking Attitudes Scale (*r* = 0.81, *p* < 0.001). Known-groups validity was supported by gender differences and associations with smoking behavior.

**Conclusion:**

The ATHS-MENA demonstrates strong psychometric properties for assessing hookah smoking attitudes in the MENA region. Its culturally sensitive design enhances its relevance and utility for research and public health interventions aimed at addressing tobacco use in this population. Future research should address sampling limitations by recruiting more balanced samples and confirm the factor structure across diverse demographic groups.

## Introduction

Smoking, whether through cigarettes, hookahs, or vaping, remains a pressing global health concern, responsible for approximately 8 million premature deaths annually, with 1.3 million attributed to secondhand smoke exposure (World Health Organization, 2018). The health consequences span cancers, cardiovascular diseases, and respiratory conditions (Haddad et al., 2016), with tobacco use killing more people daily than terrorism does in a year. Despite declining global smoking rates, the persistence of smoking-related deaths underscores the urgency of addressing this epidemic, particularly as its burden shifts toward low- and middle-income countries.

Psychologists play a critical role in understanding why people smoke, as the behavior is driven by a complex interplay of psychological, social, and biological factors (Smith, 1970). Many individuals smoke to cope with stress, anxiety, or mental health symptoms, while others are influenced by peer pressure, social norms, or nicotine addiction. The ritualistic nature of smoking and the temporary mood enhancement provided by nicotine further reinforce the habit, making cessation challenging (Secades-Villa et al., 2017). Studying these motivations can inform targeted interventions to reduce smoking initiation and support cessation efforts.

The misperception that alternatives like hookah or vaping are safer exacerbates the problem, as these methods still expose users to harmful toxins and nicotine dependence (Cavazos-Rehg et al., 2015). With hookah use rising among youth due to flavored tobacco and social appeal, and vaping gaining popularity, psychologists must also examine these trends to counteract misinformation (Berg et al., 2011). By identifying the underlying psychological drivers of smoking across all forms, researchers can develop more effective public health strategies to mitigate this global crisis.

Psychometricians have long developed standardized instruments to assess how individuals perceive and engage with smoking behaviors. Tools like the modified Cigarette Evaluation Questionnaire (mCEQ; Cappelleri et al., 2007) measure the reinforcing effects of smoking, including satisfaction, psychological reward, and aversion, providing insights into why smokers continue the habit despite known risks. Similarly, the ABOUT™ Toolbox offers a suite of validated instruments to evaluate dependence, product experience, and tobacco exposure, enabling researchers to compare combustible and smoke-free products systematically (Chrea et al., 2018). These tools are grounded in rigorous psychometric testing, ensuring reliability across diverse populations and regulatory contexts.

The Perceived Risk Instrument (PRI) further expands this framework by quantifying smokers' risk perceptions of tobacco and nicotine products, including cigarettes and reduced-risk alternatives (Salzberger and Cano, 2017). Such instruments are critical for predicting uptake and cessation behaviors, as risk perception often drives decision-making. Additionally, methods like ecological momentary assessment (EMA; Shiffman et al., 2008) and cigarette butt collection (Fix et al., 2010) address limitations of retrospective self-reports, offering real-time data on smoking patterns and toxicant exposure. These advancements highlight the field's focus on precision in measuring behavioral and cognitive aspects of smoking.

Despite this progress, gaps remain in understanding nuanced attitudes toward specific tobacco products. While existing scales like the Fagerström Test for Nicotine Dependence (FTND; Heatherton et al., 1991) and Health Action Process Approach (HAPA)-based measures assess addiction severity and cessation readiness (Schwarzer, 2016), they are primarily tailored to cigarettes. The need for specialized instruments to evaluate emerging products—such as hookah—underscores the evolving nature of tobacco use and the demand for targeted psychometric tools. This sets the stage for developing new scales to capture attitudes toward understudied smoking methods.

### Hookah smoking in the MENA region: a cultural and public health challenge

Hookah smoking in the Middle East and North Africa (MENA) region carries deep cultural and social significance, making it essential to tailor psychometric instruments to this specific context (Martinasek et al., 2011). Unlike cigarettes, hookah is often intertwined with traditions, hospitality, and communal bonding, dating back centuries in countries like Iran, Turkey, and Egypt. It is a centerpiece of social gatherings, weddings, and familial interactions, symbolizing unity and shared heritage (Momenabadi et al., 2016). Standardized smoking assessments designed for Western cigarette smokers may fail to capture these nuanced cultural dimensions, leading to inaccurate risk perceptions or behavioral predictions.

Hookah smoking, also known as waterpipe or shisha, has deep historical and cultural roots in the Middle East and North Africa (MENA) region. Unlike cigarette smoking, which has become increasingly stigmatized globally, hookah remains a symbol of hospitality, social bonding, and cultural heritage in many MENA communities (Chaouachi, 2009; Momenabadi et al., 2016; Ray, 2009). Elaborately crafted hookahs, often passed down through generations, reflect local craftsmanship and pride, reinforcing the practice as a cultural artifact rather than merely a nicotine delivery system (Chatterjee, 2022). In many communities, refusing a hookah session can be perceived as rejecting hospitality, adding social pressure that generic scales might overlook.

Social acceptance of hookah in the MENA region also contrasts sharply with global anti-smoking norms. While cigarettes face increasing stigma, hookah is frequently perceived as less harmful or even benign, a misconception perpetuated by its ritualistic and flavor-centric use (Ray, 2009). This cultural normalization necessitates instruments that measure not just dependence but also symbolic meanings—such as hookah's role in maintaining social ties or preserving traditions. Without regionally adapted scales, public health campaigns risk misaligning with local values, undermining their effectiveness (Al-Delaimy and Al-Ani, 2021; Barnett et al., 2013; Gilreath et al., 2016).

Despite its cultural significance, hookah smoking poses serious health risks. A single hookah session can expose users to higher levels of nicotine, carbon monoxide, and carcinogens than a cigarette (Haddad et al., 2016; Rezk-Hanna et al., 2019). Hookah use has been linked to respiratory diseases, cardiovascular conditions, and various cancers (Aljarrah et al., 2009; Haddad et al., 2016). The World Health Organization (2018) estimates that tobacco use, including hookah, is responsible for approximately 8 million premature deaths annually globally, with 1.3 million attributed to secondhand smoke exposure.

### The need for a culturally adapted hookah scale

Most existing smoking attitude scales have been developed and validated in Western populations, primarily focusing on cigarette smoking. Instruments such as the Fagerström Test for Nicotine Dependence (FTND; Heatherton et al., 1991), the modified Cigarette Evaluation Questionnaire (mCEQ; Cappelleri et al., 2007), and the Perceived Risk Instrument (PRI; Salzberger and Cano, 2017) provide valuable frameworks for assessing nicotine dependence and risk perceptions. Tools like the ABOUT™ Toolbox (Chrea et al., 2018) offer validated instruments to evaluate dependence, product experience, and tobacco exposure, enabling researchers to compare combustible and smoke-free products systematically. However, these scales are ill-suited for capturing the culturally specific factors that influence hookah use in the MENA region.

Standardized smoking assessments designed for Western cigarette smokers may fail to capture:

1. The cultural normalization of hookah use in social settings.
2. The role of hookah in hospitality and communal bonding.
3. The misconceptions about hookah's comparative safety due to water filtration myths.
4. The gender dynamics surrounding hookah use in different MENA communities.

The misperception that alternatives like hookah or vaping are safer exacerbates the problem, as these methods still expose users to harmful toxins and nicotine dependence (Cavazos-Rehg et al., 2015). With hookah use rising among youth due to flavored tobacco and social appeal, psychologists must examine these trends to counteract misinformation (Berg et al., 2011). By identifying the underlying psychological drivers of smoking across all forms, researchers can develop more effective public health strategies.

#### Research aims

This article aims to develop a culturally relevant (in Arabic language) scale to measure attitudes toward hookah smoking in the MENA region, where the practice holds deep social and traditional significance. The scale has been developed through a structured psychometric process, beginning with exploratory factor analysis to uncover key dimensions of attitudes, followed by confirmatory factor analysis to validate the factor structure. Once reliability and dimensionality are established, the study assesses convergent validity by examining the relationship between the new scale and existing measures of general smoking attitudes. Additionally, the study evaluates known-groups validity by analyzing how attitudes toward hookah vary across demographic and behavioral factors, such as gender, age, and smoking habits. By grounding the scale in the MENA cultural context, this research aims to provide a tool that better captures the unique drivers of hookah use in the region.

## Exploratory factor analysis

### Methods

Prior to conducting exploratory factor analysis (EFA), the scale was designed to measure attitudes toward hookah smoking in the MENA region. Drawing from existing literature (as discussed above), the scale aimed to address gaps in understanding perceptions of harm, social acceptance, and behavioral tendencies related to hookah use. Key studies informed the framework, such as findings that 70.6% of male high school students viewed hookah as more socially acceptable than cigarettes (Al-Delaimy and Al-Ani, 2021), and misconceptions about its safety persisted despite evidence of higher nicotine and toxin exposure compared to cigarettes.

The initial item pool consisted of 26 questions, structured around three theoretical factors derived from prior research. The first factor, perceived health risks, incorporated items addressing beliefs about hookah's comparative harm (e.g., filtration myths) and awareness of specific dangers like cardiovascular effects or infectious disease transmission (Aljarrah et al., 2009). The second factor, cultural and social norms, captured attitudes influenced by peer behavior and media exposure, reflecting findings that friend networks and nearby hookah cafés significantly predicted usage. The third factor, behavioral intentions and usage patterns, included scenarios assessing willingness to smoke in social settings and self-reported frequency, aligning with studies showing high intention rates among students.

The scale's development integrated validated constructs from tobacco research, such as the Theory of Reasoned Action (Hale et al., 2002; Fishbein and Yzer, 2003), and adapted methodologies from similar instruments, like the Hookah Smoker Scale (Phan et al., 2021). Items were refined through expert feedback to ensure clarity and relevance to the MENA context, where cultural embedding of hookah complicates public health messaging. This groundwork ensured the scale's readiness for empirical validation via EFA to confirm its factor structure and reliability.

#### Item development framework

The development of the Attitudes Toward Hookah Smoking in the MENA Region Scale (ATHS-MENA) followed a systematic, multi-phase process to ensure content validity and cultural appropriateness.

##### Phase 1: conceptualization and item pool generation

An initial pool of 26 items was generated based on:

1. Theoretical grounding in established health behavior models, including the Health Belief Model (HBM; Janz and Becker, 1984; Rosenstock, 2000), Theory of Planned Behavior (TPB; Ajzen, 1991; Bosnjak et al., 2020; Noar and Zimmerman, 2005; Noar et al., 2008), and Cognitive Dissonance Theory (Festinger, 1962; Joule, 1986; Joule and Beauvois, 1997; Greenwald and Ronis, 1978).
2. Literature review of existing smoking attitude scales, particularly those assessing tobacco perceptions, risk awareness, and social norms in MENA contexts. Key sources included Aljarrah et al. (2009), Martinasek et al. (2011), and Momenabadi et al. (2016).
3. Qualitative exploration of culturally specific attitudes toward hookah smoking in the Gulf region, drawing from anthropological studies of hookah's role in hospitality, communal bonding, and cultural identity (Chatterjee, 2022; Ray, 2009).
4. Expert consultation with five subject matter experts specializing in health psychology, psychometrics, and MENA public health. Experts were selected based on their publication record in tobacco research and their familiarity with the cultural context of the region. They were asked to evaluate each item for clarity, relevance, and cultural appropriateness. Items with content validity index (CVI) scores below 0.78 were revised or removed (Polit and Beck, 2006).

##### Phase 2: cognitive interviewing and pilot testing

A preliminary version of the scale (26 items) was pilot-tested with 30 participants (15 hookah users, 15 non-users) recruited from a university setting in the UAE. Participants were asked to complete the scale and then participate in cognitive interviews (Willis, 2004) to assess their comprehension of each item, including: whether the item was clearly understood; whether the item's meaning was interpreted as intended; whether any items were considered culturally inappropriate or offensive; whether response options adequately captured their perspectives.

Based on the pilot test feedback, three items were reworded for clarity, and one item was removed due to cultural sensitivity concerns. The resulting 26-item version was then subjected to empirical validation.

##### Phase 3: translation and back-translation

The final English version was translated into Arabic by two bilingual translators independently. A third translator back-translated the Arabic version into English. Discrepancies were discussed and resolved through consensus, ensuring semantic and conceptual equivalence across languages. No significant discrepancies requiring corrections were identified in the back-translation process.

#### Participants and sampling strategy

The survey was conducted with 220 participants recruited via non-probability sampling (primarily combining convenience and purposive methods) across the UAE, targeting hookah users and non-users in settings such as lounges, malls, and universities. Non-probability sampling was justified due to the exploratory nature of the study, limited resources, and the need to access hard-to-reach populations, such as frequent hookah smokers in social venues, for several reasons. First, the primary objective was instrument validation rather than population parameter estimation, where representativeness is less critical (Gabor, 2007). Similar studies in the region, like those assessing hookah practices in UAE residents, have employed this approach to efficiently gather preliminary insights despite its limitations in generalizability (Baroud et al., 2021). Second, hookah smokers, particularly frequent users, constitute a hard-to-reach population that may be less accessible through probability-based methods. Third, the diversity of recruitment settings—academic, commercial, and recreational environments—was intentionally selected to capture variation in hookah attitudes across different social contexts.

The combination of convenience and purposive sampling was implemented to maximize variation in the sample while maintaining feasibility. Purposive sampling was specifically employed to ensure inclusion of current hookah smokers, who are essential for validating the “Hookah Engagement and Dependency” factor. Similar studies in the region, like those assessing hookah practices in UAE residents, have employed this approach to efficiently gather preliminary insights (Baroud et al., 2021).

A sample size of 220 is adequate for exploratory factor analysis (EFA) with 26 items, as guidelines suggest a minimum ratio of 5–10 participants per item or an absolute minimum of 100–200 participants for stable factor solutions (de Winter et al., 2009). Our sample exceeds these thresholds, ensuring sufficient statistical power to identify the underlying factor structure of attitudes toward hookah smoking. This aligns with methodological standards in psychometric research, where comparable studies on tobacco-related scales have successfully used similar sample sizes for EFA (Ahmed et al., 2021).

#### Procedure

Participants provided demographic data (gender, age, nationality) before completing the 26-item Likert scale (1 = strongly disagree, 5 = strongly agree), with higher scores reflecting more positive attitudes toward hookah use. To mitigate response bias, negatively worded items were reverse-scored, a common practice in scale design to ensure internal consistency. The study protocol received IRB approval from Ajman University's Research Ethics Committee, M-F-H-28-June, and all participants gave informed consent, adhering to ethical guidelines for research involving human subjects.

Although the survey questions were originally designed in English, they were presented to participants in Arabic. The back-translation method was employed to ensure accuracy and cultural relevance, involving translation from English to Arabic, followed by independent translation back to English. No significant discrepancies were identified during this process, and no corrections to the Arabic version were necessary, confirming the semantic equivalence of the instrument across languages.

#### Statistical analysis

Cronbach's alpha was calculated to assess internal consistency. In the exploratory factor analysis (EFA), factors were extracted based on parallel analysis using the minimum residuals method with oblimin rotation. Parallel analysis was chosen over the Kaiser criterion or scree plot because it provides a more accurate estimation of the number of factors by comparing the eigenvalues from the sample data to those derived from random data matrices of the same size, thus reducing over-extraction. The minimum residuals method was employed to minimize the differences between observed and reproduced correlation matrices, while oblimin rotation was selected because it allows factors to correlate, which is theoretically plausible given the interconnectedness of attitudes toward hookah smoking.

Complementary methods for factor retention included:

1. Minimum Average Partial (MAP) Test: Conducted using the psych package in R to confirm the number of factors (Velicer, 1976).
2. Scree Plot Examination: Visual inspection for the point of inflection.
3. Interpretability: Assessment of theoretical meaningfulness of the factor solution.

A threshold of 0.4 was set for factor loadings to determine item retention.

#### Results

##### Participant demographics

The sample had a mean age of 25.4 years (SD = 8.65), with 79.5% female and 20.5% male participants. Nationality distribution showed that 24.5% were Emirati, while the remaining 75.5% were expatriates from other countries. The sample's demographic characteristics reflect the challenges of convenience sampling in the UAE context, where university enrollment is predominantly female.

##### Reliability

Cronbach's alpha for the 26 items of the survey was 0.94, indicating excellent internal reliability; therefore, no items were dropped on this basis.

#### Factor structure

##### Factor retention

A threshold of 0.4 was set for factor loadings to determine item retention, resulting in the removal of two items: “The benefits of hookah smoking, such as stress reduction, outweigh potential health risks” and “There is little convincing evidence that hookah smoking leads to serious health issues.” These items were dropped because they exhibited factor loadings below the 0.4 threshold on all extracted factors. Some remaining items exhibited cross-loadings, with loadings below 0.4 on one factor but above 0.4 on another. In such cases, the factor with loadings below the 0.4 threshold was discarded, resulting in a final four-factor solution. Parallel analysis, MAP test, and scree plot examination all converged on a four-factor solution. The Kaiser-Meyer-Olkin (KMO) measure of sampling adequacy was 0.940, and all measures of sampling adequacy (MSA) were above 0.82, indicating that the data were suitable for factor analysis. Bartlett's test of sphericity was significant [χ^2^_(325)_ = 4,707, *p* < 0.001], confirming that the correlation matrix was not an identity matrix and factor analysis was appropriate.

Despite the significant Bartlett's test, it's important to acknowledge that this test is often overly sensitive with large sample sizes, and deviations from sphericity are common in social science data. As such, failure to meet this assumption perfectly is a minor limitation and does not invalidate the factor analysis. The model fit indices indicated a Root Mean Square Error of Approximation (RMSEA) of 0.0636 (90% CI: 0.0541, 0.0736) and a Tucker-Lewis Index (TLI) of 0.933. While the TLI suggests acceptable fit, the RMSEA is slightly above the conventional cutoff of 0.06, indicating a marginal lack of perfect fit. However, given the complexity of attitudinal constructs and potential for model misspecification in exploratory research, these indices represent a reasonable approximation, and further refinement through confirmatory factor analysis (CFA) may address these limitations in future studies.

The scale was initially designed with a predefined four-factor structure, each representing distinct dimensions of attitudes toward hookah smoking. However, the exploratory factor analysis (EFA) revealed a different pattern in the item distribution across the four extracted factors. Although the EFA confirmed the presence of four underlying factors, the specific items loading on each factor deviated from the hypothesized structure based on the original survey design.

To better align the empirical findings with theoretical constructs and relevant literature, the decision was made to retain the four-factor structure but rename the factors to accurately reflect the observed item distribution. This process involved a careful review of the items loading on each factor, followed by a theoretical interpretation to derive meaningful and conceptually coherent labels. The resulting four dimensions were named: (1) Hookah Engagement and Dependency; (2) Social and Cultural Acceptance of Hookah; (3) Hookah Avoidance and Health Awareness; and (4) Hookah Risk Minimization. This revised framework provides a more empirically grounded and nuanced understanding of attitudes driving hookah smoking behaviors.

Items distributed along the four factors and the factor loadings for each item are presented in Table 1. The Eigenvalues plot for the Exploratory Factor Analysis is presented in Figure 1. The KMO values for each item of the EFA are presented in Table 2.

**Table 1:** Factor loadings (exploratory factor analysis).

Item	F1	F2	F3	F4	Uniqueness
I frequently smoke hookah alone for relaxation.	0.997				0.192
I often seek opportunities to smoke hookah.	0.899				0.141
I feel a strong desire to smoke hookah when socializing with friends.	0.845				0.224
I am always open to trying new flavors or types of hookah.	0.797				0.264
Hookah sessions are an important part of my leisure activities.	0.749				0.129
I plan to smoke hookah regularly in the future.	0.699				0.234
I find it difficult to imagine my social life without hookah.	0.680				0.192
Enjoying hookah with friends enhances my social experiences.	0.509	0.349			0.172
Hookah smoking should be a symbol of hospitality in my community.		0.844			0.149
Hookah smoking should be an important part of our cultural tradition.		0.770			0.378
Compared to other smoking methods, hookah is a healthier choice.		0.637			0.454
Social gatherings feel more enjoyable with the presence of a hookah.		0.607	0.430		0.172
Sharing a hookah should be a valued social experience during gatherings.	0.327	0.581			0.247
Participating in hookah sessions helps me connect with others socially.		0.560			0.204
I am not concerned about the potential health effects of hookah.		0.527			0.448
The benefits of hookah smoking, such as stress reduction, outweigh potential health risks. (removed)	0.343	0.335			0.494
I dislike it when I am pressured to smoke hookah in social situations.			0.796		0.393
People in my community should view hookah smoking as inappropriate.			0.766		0.402
I usually avoid situations where hookah is being smoked.			0.726		0.396
In my culture, hookah smoking should be frowned upon.			0.712		0.497
I intend to not smoke hookah in the coming months.			0.632		0.506
Hookah smoke contains harmful toxins that can damage my health.			0.502		0.554
Regular hookah smoking can lead to serious health problems.			0.473		0.623
When used occasionally, hookah poses little threat to my health.				0.792	0.378
Hookah smoking is a relaxing activity with minimal health risks.				0.608	0.539
There is little convincing evidence that hookah smoking leads to serious health issues. (removed)				0.393	0.683

F1, hookah engagement and dependency; F2, social and cultural acceptance; F3, hookah avoidance and health awareness; F4, hookah risk minimization. Items marked (removed) were dropped for loadings below the 0.40 threshold.

**Figure 1:**
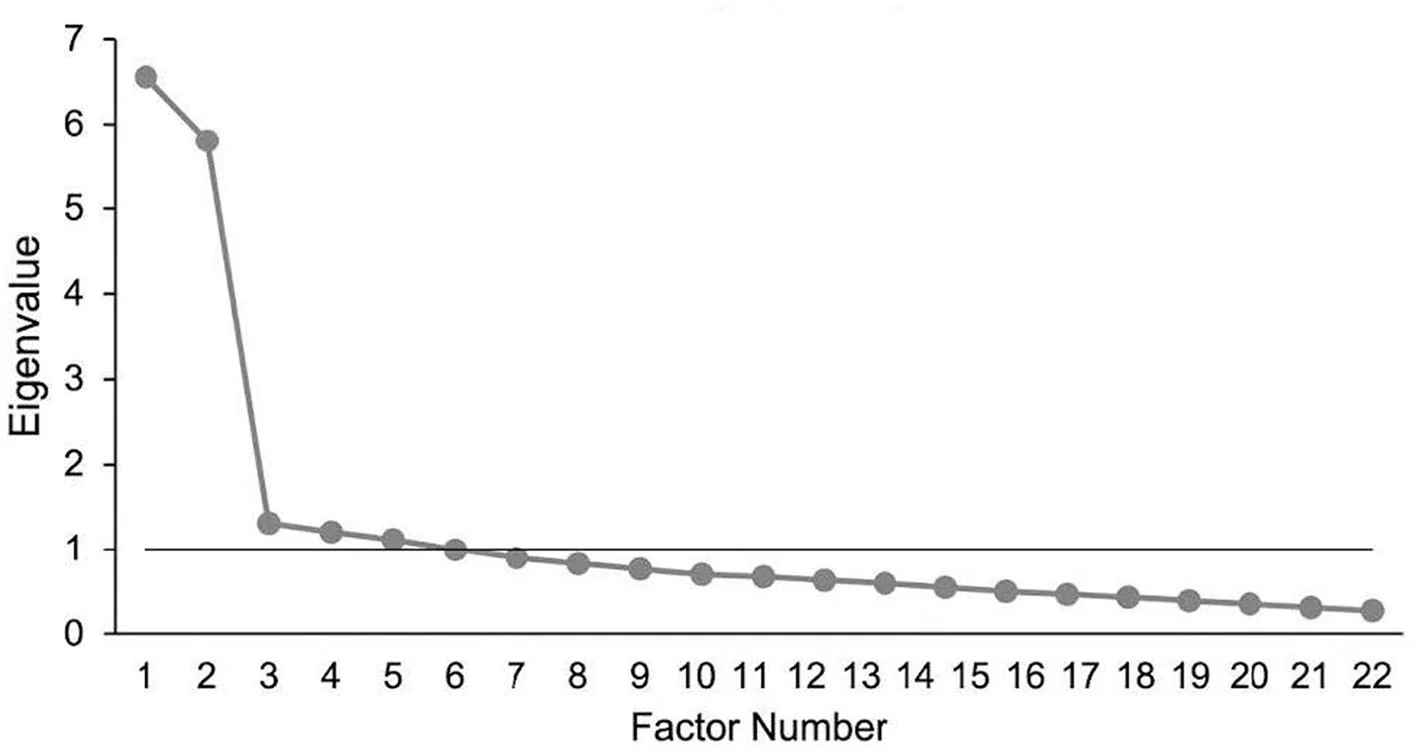
Eigenvalues scree plot for exploratory factor analysis.

**Table 2:** Item measures of sampling adequacy (MSA)—exploratory factor analysis.

Metric/parameter	Value/description
Overall KMO	0.940
Individual Item MSA range	0.82–0.97
Recommended threshold	0.60
Interpretation	All values exceed the threshold, confirming data suitability for factor analysis.

##### Item retention

A threshold of 0.4 was set for factor loadings to determine item retention, resulting in the removal of two items: “The benefits of hookah smoking, such as stress reduction, outweigh potential health risks” and “There is little convincing evidence that hookah smoking leads to serious health issues.” These items were dropped because they exhibited factor loadings below the 0.4 threshold on all extracted factors. Some remaining items exhibited cross-loadings, with loadings below 0.4 on one factor but above 0.4 on another. In such cases, the factor with loadings below the 0.4 threshold was discarded, resulting in a final four-factor solution.

#### Total variance explained

The final four-factor solution explained 68.4% of the total variance in attitudes toward hookah smoking, with the following factor-specific explained variances (Table 3).

**Table 3:** Variance explained for the four-factor solution of the ATHS-MENA scale.

Factor	Label	Explained variance (%)
1	Hookah engagement and dependency	29.7
2	Social and cultural acceptance	18.2
3	Hookah avoidance and health awareness	11.5
4	Hookah risk minimization	9.0

#### Factor inter-correlations

The inter-factor correlations were moderate to strong, supporting the theoretical interconnectedness of the dimensions (Table 4).

**Table 4:** Inter-factor correlations for the ATHS-MENA dimensions.

Factors	*r*	*p*-value
Factor 1 ↔ Factor 2	0.57	< 0.001
Factor 1 ↔ Factor 3	−0.41	< 0.001
Factor 1 ↔ Factor 4	0.48	< 0.001
Factor 2 ↔ Factor 3	−0.36	< 0.001
Factor 2 ↔ Factor 4	0.52	< 0.001
Factor 3 ↔ Factor 4	−0.31	< 0.001

Factor 1, hookah engagement and dependency; Factor 2, social and cultural acceptance; factor 3, hookah avoidance and health awareness; Factor 4, hookah risk minimization.

#### Factor renaming and interpretation

The scale was initially designed with a hypothesized four-factor structure based on theoretical considerations. However, the exploratory factor analysis (EFA) revealed a different pattern in the item distribution across the four extracted factors. Although the EFA confirmed the presence of four underlying factors, the specific items loading on each factor deviated from the hypothesized structure based on the original survey design.

To better align the empirical findings with theoretical constructs and relevant literature, the decision was made to retain the four-factor structure but rename the factors to accurately reflect the observed item distribution. This process involved a careful review of the items loading on each factor, followed by a theoretical interpretation to derive meaningful and conceptually coherent labels. The resulting four dimensions were named: (1) Hookah Engagement and Dependency; (2) Social and Cultural Acceptance of Hookah; (3) Hookah Avoidance and Health Awareness; and (4) Hookah Risk Minimization. This revised framework provides a more empirically grounded and nuanced understanding of attitudes driving hookah smoking behaviors.

## Confirmatory factor analysis

### Methods

#### Participants

For the confirmatory factor analysis (CFA), we surveyed 247 participants (different from the exploratory factor analysis, as they were sampled from a different pool) who completed the Arabic version of our questionnaire, which included both the retained Likert-scale items from the exploratory factor analysis (EFA) and demographic questions about age, gender, ethnicity (Arab vs. non-Arab), nationality (Emirati vs. non-Emirati). This sample size is well within the recommended range for CFA, as studies indicate that 200–300 participants are generally sufficient for validating a four-factor model, particularly when factor loadings are moderate to strong—a condition met in our EFA, where all retained items loaded above 0.4. The demographic diversity of the sample, with representation across key social variables (gender, nationality, ethnicity), helps ensure that the factor structure is tested under varied conditions, enhancing the robustness of our validity assessments.

While we used non-probability sampling, this approach was appropriate for the study's focus on scale validation rather than population generalization. The inclusion of detailed demographic variables allowed us to examine potential subgroup differences in how the four factors—Hookah Engagement and Dependency, Social and Cultural Acceptance, Hookah Avoidance and Health Awareness, and Hookah Risk Minimization—operate across distinct segments of the sample. This design prioritizes internal validity, ensuring that the measure performs consistently among groups with differing backgrounds. Moreover, the high internal reliability (α = 0.94) and careful translation process (verified through back-translation with no modifications needed) further support the suitability of the data for CFA.

#### Sample characteristics

Participants had a mean age of 22.8 (SD = 6.72). 89.1% were female; the rest male. 23.5% were Emirati; the rest were expatriates; 83.8% self-identified as Arabs. The demographic diversity of the sample, with representation across key social variables (gender, nationality, ethnicity), helps ensure that the factor structure is tested under varied conditions, enhancing the robustness of our validity assessments.

#### Model specification and fit evaluation

The CFA aimed to confirm the factor structure identified in the EFA. Although the non-probability sampling limits broader inferences, the sample's heterogeneity provides a solid foundation for evaluating whether the measure behaves as expected across key demographic divides. Future research could expand on these findings with probability-based samples, but the current analysis establishes a strong basis for the scale's structural validity and sets the stage for more nuanced investigations into how attitudes toward hookah smoking vary by age, nationality, socioeconomic status, and other contextual factors.

All items retained from the EFA demonstrated significant factor loadings (*p* < 0.05), indicating that each item significantly contributed to its respective factor. Consequently, all items were preserved in the model, aligning with the four dimensions originally obtained in the EFA: Hookah Engagement and Dependency, Social and Cultural Acceptance of Hookah, Hookah Avoidance and Health Awareness, and Hookah Risk Minimization.

#### Model modification

Following the initial CFA, model fit was improved through the addition of theoretically justified correlated residuals between conceptually related items. Specifically, error terms were allowed to correlate between items measuring:

- Health risk knowledge (Items 19 and 21).
- Behavioral avoidance (Items 20 and 22).
- Cultural acceptance (Items 16 and 17).
- Personal engagement (Items 9 and 4).

These modifications were guided by modification indices and theoretical considerations to avoid capitalization on chance. Additional model refinement involved removing items with standardized factor loadings below 0.40. Items “Hookah smoke contains harmful toxins that can damage my health” (λ = 0.322) and “Regular hookah smoking can lead to serious health problems” (λ = 0.373) were removed due to their weak contribution to the “Hookah Avoidance and Health Awareness” factor.

### Results

Participants had a mean age of 22.8, SD = 6.72. 89.1% were female; the rest male. 23.5% were Emirati; the rest were expatriates; 83.8% self-identified as Arabs.

All items retained from the EFA demonstrated significant factor loadings (*p* < 0.05), indicating that each item significantly contributed to its respective factor. Factor loadings of each item are presented in Table 5, the path diagram of the analysis is presented in Figure 2. Consequently, all items were preserved in the model, aligning with the four dimensions originally obtained in the EFA: Hookah Engagement and Dependency, Social and Cultural Acceptance of Hookah, Hookah Avoidance and Health Awareness, and Hookah Risk Minimization.

**Table 5:** Confirmatory factor analysis (CFA) model fit indices & factor loadings.

Model fit indices			
Index	Value	Threshold	Interpretation
Chi-square (χ^2^)	503 (*df* = 185)	—	Significant (*p* < 0.001)
CFI	0.912	≥ 0.90	Acceptable
TLI	0.901	≥ 0.90	Acceptable
RMSEA	0.079	< 0.08	Acceptable
RMSEA 90% CI	0.071, 0.087	—	—
SRMR	0.062	< 0.08	Acceptable
Factor loadings (confirmatory factor analysis)			
Factor	Item	Estimate	*p*
Hookah risk minimization	When used occasionally, hookah poses little threat to my health.	0.86	< 0.001
Hookah risk minimization	Hookah smoking is a relaxing activity with minimal health risks.	0.79	< 0.001
Hookah engagement and dependency	I feel a strong desire to smoke hookah when socializing with friends.	0.73	< 0.001
Hookah engagement and dependency	Enjoying hookah with friends enhances my social experiences.	0.74	< 0.001
Hookah engagement and dependency	I find it difficult to imagine my social life without hookah.	0.68	< 0.001
Hookah engagement and dependency	I plan to smoke hookah regularly in the future.	0.66	< 0.001
Hookah engagement and dependency	Hookah sessions are an important part of my leisure activities.	0.76	< 0.001
Hookah engagement and dependency	I am always open to trying new flavors or types of hookah.	0.74	< 0.001
Hookah engagement and dependency	I often seek opportunities to smoke hookah.	0.75	< 0.001
Hookah engagement and dependency	I frequently smoke hookah alone for relaxation.	0.69	< 0.001
Social and cultural acceptance	I am not concerned about the potential health effects of hookah.	0.63	< 0.001
Social and cultural acceptance	Participating in hookah sessions helps me connect with others socially.	0.76	< 0.001
Social and cultural acceptance	Sharing a hookah should be a valued social experience during gatherings.	0.70	< 0.001
Social and cultural acceptance	Social gatherings feel more enjoyable with the presence of a hookah.	0.79	< 0.001
Social and cultural acceptance	Compared to other smoking methods, hookah is a healthier choice.	0.62	< 0.001
Social and cultural acceptance	Hookah smoking should be an important part of our cultural traditions.	0.67	< 0.001
Social and cultural acceptance	Hookah smoking should be a symbol of hospitality in my community.	0.72	< 0.001
Hookah avoidance and health awareness	In my culture, hookah smoking should be frowned upon.	0.62	< 0.001
Hookah avoidance and health awareness	I usually avoid situations where hookah is being smoked.	0.58	< 0.001
Hookah avoidance and health awareness	I intend to not smoke hookah in the coming months.	0.52	< 0.001
Hookah avoidance and health awareness	I dislike it when I am pressured to smoke hookah in social situations.	0.53	< 0.001
Hookah avoidance and health awareness	People in my community should view hookah smoking as inappropriate.	0.51	< 0.001

**Figure 2:**
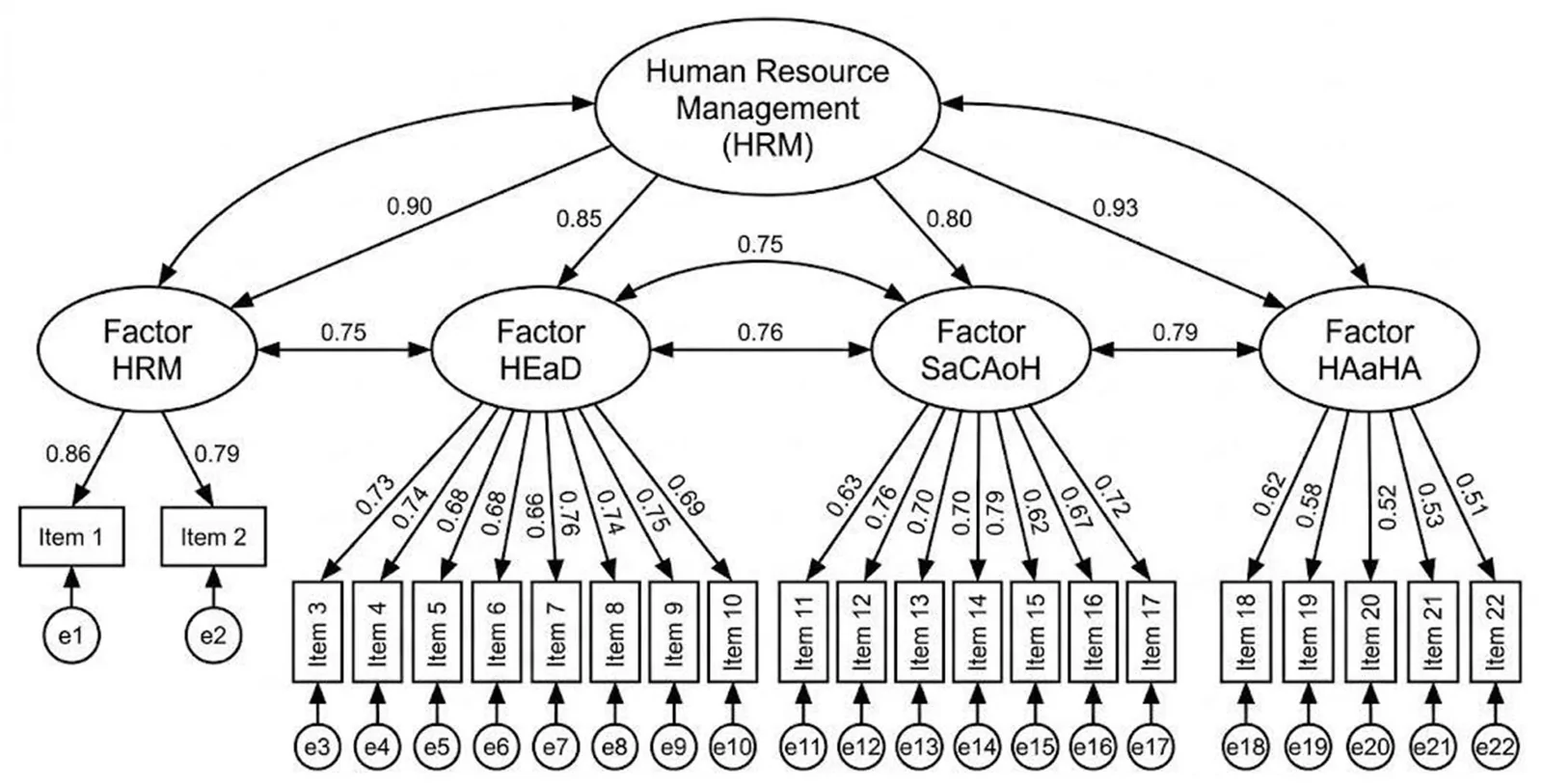
Path diagram for confirmatory factor analysis. HRM, hookah risk minimization; HEaD, hookah engagement and dependence; SaCAoH, social and cultural acceptance of hookah; HAaHA, hookah avoidance and health awareness.

#### Model fit

The model fit indices for the revised model revealed in Table 6. The standardized factor loadings and reliability coefficients for each item and factor are presented in Table 7.

**Table 6:** Confirmatory factor analysis (CFA) model fit indices.

Index	Value	Threshold	Interpretation
Chi-square	503	—	Significant (*p* < 0.001)
*df*	185	—	—
CFI	0.912	≥0.90	Acceptable
TLI	0.901	≥0.90	Acceptable
RMSEA	0.079	< 0.08	Acceptable
RMSEA 90% CI	0.071, 0.087	—	—
SRMR	0.062	< 0.08	Acceptable

**Table 7:** Standardized factor loadings for the confirmatory factor analysis (CFA) model.

Factor	Item	Estimate	*p*
Hookah risk minimization	When used occasionally, hookah poses little threat to my health.	0.86	< 0.001
	Hookah smoking is a relaxing activity with minimal health risks.	0.79	< 0.001
Hookah engagement and dependency	I feel a strong desire to smoke hookah when socializing with friends.	0.73	< 0.001
	Enjoying hookah with friends enhances my social experiences.	0.74	< 0.001
	I find it difficult to imagine my social life without hookah.	0.68	< 0.001
	I plan to smoke hookah regularly in the future.	0.66	< 0.001
	Hookah sessions are an important part of my leisure activities.	0.76	< 0.001
	I am always open to trying new flavors or types of hookah.	0.74	< 0.001
	I often seek opportunities to smoke hookah.	0.75	< 0.001
	I frequently smoke hookah alone for relaxation.	0.69	< 0.001
Social and cultural acceptance	I am not concerned about the potential health effects of hookah.	0.63	< 0.001
	Participating in hookah sessions helps me connect with others socially.	0.76	< 0.001
	Sharing a hookah should be a valued social experience during gatherings.	0.70	< 0.001
	Social gatherings feel more enjoyable with the presence of a hookah.	0.79	< 0.001
	Compared to other smoking methods, hookah is a healthier choice.	0.62	< 0.001
	Hookah smoking should be an important part of our cultural traditions.	0.67	< 0.001
	Hookah smoking should be a symbol of hospitality in my community.	0.72	< 0.001
Hookah avoidance and health awareness	In my culture, hookah smoking should be frowned upon.	0.62	< 0.001
	I usually avoid situations where hookah is being smoked.	0.58	< 0.001
	I intend to not smoke hookah in the coming months.	0.52	< 0.001
	I dislike it when I am pressured to smoke hookah in social situations.	0.53	< 0.001
	People in my community should view hookah smoking as inappropriate.	0.51	< 0.001

While the Chi-square test was significant, this test is known to be sensitive to sample size, often leading to rejection of the model even with reasonable fit. The CFI and TLI values now meet the generally accepted threshold of 0.90, and the RMSEA value is at the cutoff of 0.08, indicating acceptable fit.

Despite these fit statistics, we opted to retain the model due to several considerations. First, the EFA provided strong support for the four-factor structure, and the CFA confirmed the significant loadings of all items on their respective factors. Second, given the complexity of measuring attitudes and the potential for model misspecification, especially in cross-sectional data, achieving perfect fit is often unrealistic. Third, the model provides a theoretically meaningful and interpretable framework for understanding attitudes toward hookah smoking, consistent with previous literature. Therefore, while acknowledging the limitations in model fit, we believe that the current model offers valuable insights into the underlying dimensions of hookah attitudes and justifies its use for subsequent analyses.

## Convergent validity

### Methods

To examine convergent validity, we administered the validated Smoking Attitudes Scale (SAS) by Shore et al. (2000) alongside our ATHS-MENA scale to the same 247 participants. The SAS has demonstrated strong reliability (α > 0.80) in prior studies. Some of the items are reverse-scored. Higher SAS scores indicate more positive attitudes toward smoking. One specific item was excluded from the survey for cultural sensitivity reasons in the MENA region. We computed total scores for both scales, as well as for each of the scales' dimensions, and assessed their associations (Spearman's rank) to determine whether general smoking attitudes (measured by SAS) aligned with overall attitudes toward hookah (measured by ATHS-MENA).

### Results

The Spearman correlation between the total scores of the two scales was strong (*r* = 0.81, *p* < 0.001), indicating a significant and positive relationship. This result supports the convergent validity of our scale, as it aligns well with an established measure of attitudes toward smoking. The strong correlation suggests that both scales capture similar underlying constructs related to smoking behaviors and perceptions. The matrix of correlations between the dimensions of our hookah attitude scale and those of the SAS, presented in Table 8, revealed consistent and significant associations across corresponding dimensions, further reinforcing the theoretical alignment between the two measures. However, the high correlation coefficient also raises a question about discriminant validity, implying that the ATHS-MENA may share considerable overlap with general smoking attitudes. Future research should investigate the discriminant validity of the ATHS-MENA relative to other measures of health risk behaviors.

**Table 8:** Spearman coefficients for correlation between dimensions of attitudes toward hookah in the MENA region and general attitudes toward smoking.

ATHS-MENA dimensions	5. Relationships with smokers	6. Views toward smoking	7. Concerns about secondhand smoke	8. Attitudes concerning hookah
1. Hookah engagement and dependency	0.645^**^	0.632^**^	0.354^**^	0.553^**^
2. Social and cultural aspects	0.610^**^	0.634^**^	0.337^**^	0.521^**^
3. Hookah avoidance	0.430^**^	0.361^**^	0.545^**^	0.580^**^
4. Hookah risk minimization	0.366^**^	0.436^**^	0.221^**^	0.276^**^

^**^p < 0.001. 5, relationships with smokers; 6, views toward smoking; 7, concerns about secondhand smoke; 8, attitudes concerning hookah.

## Known-groups validity

### Methods

To assess known-groups validity, we utilized the same sample of participants (n = 247) who were included in the confirmatory factor analysis and convergent validity assessments. This approach allowed us to evaluate whether our hookah attitude scale could effectively differentiate between groups with theoretically distinct characteristics. Specifically, we compared participants' scores on the scale across key demographic and behavioral variables, including gender, nationality (Emirati vs. non-Emirati), ethnicity (Arab vs. non-Arab), frequency of hookah smoking, average number of vaping puffs per day, and average number of cigarettes smoked per day.

For the ordinal variables of frequency of hookah smoking, average number of vaping puffs per day, and average number of cigarettes smoked per day, each value was converted into ranks to facilitate statistical analysis. All behavioral variables were then tested for association with the total score of our newly designed hookah attitude scale using Spearman's rank correlation coefficient.

To compare genders, ethnicity and nationality, we used Mann-Whitney U tests, as each of these variables compared two groups, and the variables are based at the ordinal level of measurement; consequently, the tests should be non-parametric. All statistical analyses were conducted using Jamovi, with statistical significance set at *p* < 0.05.

### Results

Spearman's rank correlation analysis revealed several significant associations between the total score on our hookah attitude scale (“Hookah Total”) and the numerical variables, as detailed in Table 9. A notable positive correlation was observed between the total score and frequency of hookah smoking (*r* = 0.475, *p* < 0.001), indicating that individuals who smoke hookah more frequently tend to have more positive attitudes toward it. Similarly, positive associations were found between the total score and both the number of vaping puffs per day (*r* = 0.404, *p* < 0.001) and the number of cigarettes smoked per day (*r* = 0.278, *p* < 0.001), suggesting that individuals who vape or smoke cigarettes more frequently also hold more positive attitudes toward hookah.

**Table 9:** Spearman coefficients of associations with attitudes toward hookah smoking in the MENA region.

Variable	*r*
Frequency in hookah smoking	0.475^***^
Number of vaping puffs per day	0.404^***^
Number of cigarettes per day	0.278^***^
Financial status	0.040
Age	0.095

^***^p < 0.001.

Mann-Whitney *U* test revealed a significant difference between females (median = 38.5) and males (median = 44.0; *U* = 2,234, *p* = 0.036) in the values for the designed scale. No significant differences were observed between Arabs (median = 40.0) and non-Arabs (median = 45.0; *U* = 3762, *p* = 0.361), nor between Emiratis (median = 37.0) and non-Emiratis (median = 41.0; *U* = 4948, *p* = 0.263).

## Discussion

The act of smoking is far from monolithic; it encompasses a range of methods each with distinct cultural associations and perceived risks (Day et al., 2014; Hoeppner et al., 2017; Lowenthal, 2015; Lujic et al., 2005). While cigarette smoking faces increasing stigmatization, particularly in Western contexts, hookah smoking has carved out a unique space, especially among younger individuals. This phenomenon warrants investigation beyond simple risk assessments, requiring an understanding of the motivations driving this specific form of tobacco use.

Hookah's ascendance in popularity, most notably in the MENA region, presents a compelling divergence from global anti-smoking trends. Often perceived as a socially acceptable pastime, hookah is interwoven with traditions of hospitality and communal gatherings (Barnett et al., 2013; Chaouachi, 2009; Gilreath et al., 2016; Rezk-Hanna et al., 2019). This perception contrasts sharply with the image of cigarettes, particularly among young adults who may view hookah as a less harmful or more socially desirable alternative, highlighting the need for nuanced understanding of these perceptions.

### Factor structure and theoretical implications

The resulting four-factor structure of the ATHS-MENA scale aligns with established theories of health behavior and risk perception. The factors represent distinct dimensions of attitudes toward hookah smoking, encompassing beliefs about its health consequences, social acceptability, personal intentions, and perceived addictiveness. This multi-dimensional approach is consistent with models such as the Health Belief Model (Green et al., 2020; Janz and Becker, 1984; Orji et al., 2012; Rosenstock, 2000; Sheeran and Abraham, 1996) and the Theory of Planned Behavior, which posit that attitudes, subjective norms, and perceived behavioral control all contribute to health-related decisions (Bosnjak et al., 2020; Godin and Kok, 1996; Sheeran et al., 2001; Sniehotta, 2009).

#### Hookah engagement and dependency

The emergence of “Hookah Engagement and Dependency” as a distinct factor aligns with addiction theories that emphasize the reinforcing properties of nicotine and the development of compulsive behaviors (Tiwari et al., 2020). This dimension captures the extent to which individuals experience cravings, prioritize hookah smoking in their daily routines, and struggle to reduce their consumption, reflecting core elements of addiction as defined by diagnostic criteria such as those in the DSM-5 (Chou et al., 2016). It moves beyond simple usage frequency to tap into the psychological and physiological aspects of dependence.

#### Social and cultural acceptance

The “Social and Cultural Acceptance of Hookah” factor reflects the profound influence of social norms and cultural values on health behaviors, particularly relevant in the MENA region. This dimension captures the degree to which individuals perceive hookah smoking as a socially acceptable and culturally valued activity, influenced by factors such as peer behavior, family traditions, and media portrayals. This resonates with social cognitive theory (Van Zundert et al., 2009), highlighting how observational learning and social reinforcement contribute to the adoption and maintenance of hookah smoking habits.

#### Hookah avoidance and health awareness

The “Hookah Avoidance and Health Awareness” factor underscores the role of health beliefs and risk perceptions in shaping attitudes toward hookah smoking. This dimension captures the extent to which individuals are aware of the health risks associated with hookah smoking and are motivated to avoid or reduce their consumption based on these concerns. This aligns with the Health Belief Model, which posits that perceived susceptibility to illness and perceived severity of consequences are key determinants of health-related behaviors (Sotodeh et al., 2016). It also reflects the impact of public health campaigns and educational initiatives on promoting health awareness.

#### Hookah risk minimization: a potential explanation

The “Hookah Risk Minimization” factor may be understood through the lens of cognitive dissonance theory, though it is important to note that we did not directly measure cognitive dissonance in this study. Cognitive dissonance theory (Festinger, 1962) suggests that individuals experience psychological discomfort when their behavior conflicts with their beliefs. When individuals smoke hookah despite awareness of health risks, they may reduce this discomfort through cognitive strategies such as minimizing the perceived severity of risks or questioning the validity of risk information (McGrath, 2017).

The items loading on this factor—such as “When used occasionally, hookah poses little threat to my health” and “Hookah smoking is a relaxing activity with minimal health risks”—reflect such risk minimization strategies. These items may represent attempts to justify continued hookah use by downplaying its potential negative consequences. However, this interpretation remains theoretical and should be treated as a plausible explanation rather than a confirmed mechanism. Future research employing experimental or longitudinal designs is needed to establish whether cognitive dissonance processes causally drive risk minimization in hookah smokers.

Alternative explanations for this factor should also be considered. For instance, the factor may reflect genuine differences in risk perception, with some individuals having less accurate knowledge about hookah's health effects. This is consistent with the Health Belief Model, where perceived severity of health consequences is a key determinant of behavior (Rosenstock, 2000). The factor could also reflect optimism bias (Weinstein, 1989), where individuals believe they are less susceptible to harm than others. We encourage future research to test these competing explanations directly.

#### Convergent validity

The results of this study demonstrate that convergent validity was achieved for the ATHS-MENA. The scale showed a significant correlation with an important existing measure of general smoking attitudes. This indicates that the ATHS-MENA is measuring a similar underlying construct to other established smoking attitude scales, but with a specific focus on the nuances of hookah smoking within the MENA region.

Convergent validity was achieved to the extent that, despite the unique aspects of hookah smoking, it shares fundamental psychological underpinnings with other forms of smoking. Theoretically, this finding is grounded in the broader understanding of attitude formation and behavior: attitudes toward specific behaviors, like smoking, are often related to more general attitudes toward the overall category of the behavior.

However, the very high correlation (*r* = 0.81) suggests the scales may share considerable overlap in the constructs they measure. While this confirms that the ATHS-MENA is successfully capturing general smoking attitudes, it also raises a question about discriminant validity, implying that the ATHS-MENA may not be capturing a sufficiently unique construct. Future work should investigate the discriminant validity of the ATHS-MENA relative to other measures of health risk behaviors.

#### Known-groups validity

The results also suggest that the ATHS-MENA demonstrates strong known-groups validity, indicating that it effectively differentiates between groups known to differ in their attitudes and behaviors related to hookah smoking. This suggests that individuals who report higher levels of engagement with hookah, vaping, or cigarettes also tend to score higher on specific subscales of the ATHS-MENA, reflecting a more positive attitude toward hookah smoking.

#### Gender differences

The known-groups validity of the ATHS-MENA is substantially strengthened by the significant gender differences observed in the study. The results indicate that in this sample, men exhibited higher average scores on the ATHS-MENA compared to women. This finding aligns with existing research and cultural understanding within the MENA region, where hookah smoking is often more socially accepted and prevalent among men (Aljarrah et al., 2009; Hamadeh et al., 2020; Shiraly and Mohamadinezhad, 2024).

The higher prevalence of hookah smoking among men compared to women can be attributed to several cultural, social, and psychological factors. In many societies, particularly in the MENA region, smoking hookah is often associated with masculinity and traditional gender roles, making it more socially acceptable for men to engage in this behavior (Primack et al., 2016). Additionally, peer pressure and group dynamics play a significant role, as hookah smoking is frequently a communal activity that fosters camaraderie and social bonding among men (Davids et al., 2022).

#### Nationality and citizenship

Interestingly, the study did not find significant differences in ATHS-MENA scores based on nationality (Arab vs. non-Arab) or citizenship in the UAE (Emirati vs. non-Emirati). While known-groups validity was supported by gender differences and associations with smoking behavior, the lack of differentiation based on these specific demographic variables warrants further consideration.

One possible explanation for the lack of significant differences based on nationality and Emirati status is the increasing globalization and cultural exchange within the UAE (Baburajan, 2011). It is plausible that exposure to hookah culture is becoming increasingly widespread, regardless of one's specific national or ethnic background. The absence of significant differences based on these demographic variables does not necessarily invalidate the known-groups validity of the ATHS-MENA.

## Limitations

### Sampling limitations

A significant limitation of this study is the substantial gender imbalance in both samples, with female participants comprising 79.5% (EFA) and 89.1% (CFA) of the samples. This overrepresentation of females is a common challenge in university-based convenience sampling in the UAE, where female enrollment in higher education significantly outnumbers male enrollment (Godwin, 2006; Ridge et al., 2019). While this imbalance reflects the demographic reality of the sampling context, it limits the generalizability of findings to the broader male population in the MENA region.

Importantly, the gender differences observed in this study—with males scoring higher on positive attitudes toward hookah—should be interpreted cautiously. To address this concern, we conducted supplementary analyses within the female subsample, which confirmed the four-factor structure (CFI = 0.891, TLI = 0.879, RMSEA = 0.091), suggesting that the factor structure is largely consistent even when restricting to the dominant gender group.

Future research should prioritize the recruitment of more balanced samples to confirm the factor structure and gender differences across genders. Researchers may consider targeted recruitment strategies in male-dominated settings (e.g., workplaces, cafes) or the use of quota sampling to ensure adequate male representation.

### Generalizability concerns

The use of non-probability sampling, while appropriate for this instrument validation study, limits the generalizability of findings beyond the sampled population. Specifically, the sample is predominantly composed of urban, educated youth, which may not represent the broader MENA population, including rural residents, older adults, or individuals with lower educational attainment. To address this limitation, researchers should consider stratified sampling in future studies to ensure adequate representation across age groups, educational levels, and geographic regions. Future research could employ respondent-driven sampling (RDS) or venue-based time-space sampling to recruit more representative samples of hookah users (Blebu et al., 2018; Shedlin et al., 2012).

### Measurement limitations

This study relied on self-reported measures, and this is an important limitation. While the reliance on self-report measures introduces the potential for social desirability bias, this is a common challenge in attitudinal research and efforts were made to mitigate this through reverse-scored items and ensuring anonymity. The cross-sectional design also limits our ability to infer causality, but it provides a valuable snapshot of attitudes at a specific point in time, which can inform future longitudinal investigations.

### Geographic scope

Furthermore, while the study focused on the UAE, which offers a rich and diverse cultural context within the Gulf region, it is important to recognize that nuances in hookah smoking practices may exist across other countries in the broader MENA region. However, the strong psychometric properties of the ATHS-MENA and its demonstrated validity provide confidence in its potential for adaptation and use in diverse populations. Future research could explore the scale's applicability and cultural relevance in other MENA countries, further strengthening its generalizability.

### Model fit considerations

The model fit of the initial CFA was less than ideal, requiring *post-hoc* modifications including correlated residuals and item removal. While these modifications improved fit, they may capitalize on sample-specific characteristics. Future research should cross-validate the final factor structure in independent samples.

## Conclusion

This study successfully developed and validated the Attitudes Toward Hookah Smoking Scale - MENA (ATHS-MENA), a culturally relevant instrument for measuring attitudes toward hookah smoking in the Middle East and North Africa region. Through rigorous psychometric testing, including exploratory and confirmatory factor analyses, the ATHS-MENA demonstrated strong reliability and validity, capturing key dimensions of attitudes toward hookah smoking within this unique cultural context. The scale's four-factor structure—Hookah Engagement and Dependency, Social and Cultural Acceptance of Hookah, Hookah Avoidance and Health Awareness, and Hookah Risk Minimization—provides a nuanced understanding of the multifaceted nature of these attitudes.

The ATHS-MENA's demonstrated convergent validity with existing measures of general smoking attitudes and known-groups validity based on gender and smoking behavior further supports its utility as a research tool. These findings suggest that the ATHS-MENA not only measures a construct related to general smoking attitudes but also differentiates between groups with varying levels of engagement with hookah smoking. This makes it a valuable tool for researchers and public health professionals seeking to understand and address hookah smoking in the MENA region.

The global health concern regarding smoking necessitates understanding the nuances of various methods, especially in regions where alternative methods like hookah hold cultural significance. Gaps remain in understanding nuanced attitudes toward specific tobacco products; the ATHS-MENA addresses this gap by providing a culturally sensitive instrument specifically designed for the MENA region.

Consequently, the ATHS-MENA represents a significant advancement in the assessment of attitudes toward hookah smoking in the MENA region. Its robust psychometric properties and cultural relevance make it a valuable tool for future research, public health interventions, and policy development. Further studies should explore the scale's predictive validity and its applicability in diverse populations within the MENA region.

## Data Availability

The raw data supporting the conclusions of this article will be made available by the authors, without undue reservation.

## Appendix 1

### The “attitudes toward hookah smoking in the MENA region” scale (ATHS-MENA)

Please express your level of agreement with each of these statements (Strongly disagree, Disagree, Neutral, Agree, Strongly Agree)

When used occasionally, hookah poses little threat to my health.

لصحتي بسيطاً تهديداً الشيشة تمثل لآخر، حين من استخدامها عند

Hookah smoking is a relaxing activity with minimal health risks.

ضئيلة صحية مخاطر على وينطوي مريح نشاط هو الشيشة تدخين

I feel a strong desire to smoke hookah when socializing with friends.

الأصدقاء مع الاجتماع عند الشيشة لتدخين قوية برغبة أشعر

Enjoying hookah with friends enhances my social experiences.

الاجتماعية تجاربي من يعزز الأصدقاء مع بالشيشة الاستمتاع

I find it difficult to imagine my social life without hookah.

الشيشة بدون الاجتماعية حياتي تخيل الصعب من

I plan to smoke hookah regularly in the future.

المستقبل في بانتظام الشيشة لتدخين أخطط

Hookah sessions are an important part of my leisure activities.

الترفيهية أنشطتي من مهماً جزءاً تشكل الشيشة جلسات

I am always open to trying new flavors or types of hookah.

الشيشة من جديدة أنواع أو نكهات لتجربة منفتح دائماً أنا

I often seek opportunities to smoke hookah.

الشيشة لتدخين فرص عن أبحث ما غالباً

I frequently smoke hookah alone for relaxation.

للاسترخاء بمفردي الشيشة أدخن ما غالباً

I am not concerned about the potential health effects of hookah.

الشيشة لتدخين المحتملة الصحية التأثيرات بشأن بالقلق أشعر ا

Participating in hookah sessions helps me connect with others socially.

الآخرين مع الاجتماعي التواصل على تساعدني الشيشة جلسات في المشاركة

Sharing a hookah should be a valued social experience during gatherings.

التجمعات أثناء قيمة اجتماعية تجربة الشيشة مشاركة تكون أن يجب

Social gatherings feel more enjoyable with the presence of a hookah.

الشيشة وجود مع متعة أكثر تكون الاجتماعية التجمعات

Compared to other smoking methods, hookah is a healthier choice.

صحة أكثر خيار هي الشيشة الأخرى، التدخين بوسائل مقارنة

Hookah smoking should be an important part of our cultural traditions.

الثقافية تقاليدنا من مهماً جزءاً الشيشة تدخين يكون أن يجب

Hookah smoking should be a symbol of hospitality in my community.

مجتمعي في للضيافة رمزاً الشيشة تدخين يكون أن يجب

In my culture, hookah smoking should be frowned upon.

مرفوض أمر أنه على ثقافتي في الشيشة تدخين يكون أن يجب

Hookah smoke contains harmful toxins that can damage my health.

صحتي على تؤثر قد ضارة سموم على يحتوي الشيشة دخان

I usually avoid situations where hookah is being smoked.

الشيشة تدخين فيها يتم التي المواقف أتجنب ما عادةً

Regular hookah smoking can lead to serious health problems.

خطيرة صحية مشاكل إلى يؤدي أن يمكن للشيشة المنتظم التدخين

I intend to not smoke hookah in the coming months.

القادمة الأشهر في الشيشة تدخين عدم أنوي

People in my community should view hookah smoking as inappropriate.

لائق غير أمر أنه على الشيشة تدخين مجتمعي في الناس يرى أن يجب

I dislike it when I am pressured to smoke hookah in social situations.

الاجتماعية المواقف في الشيشة لتدخين علي الضغط يتم أن أحب لا
